# Recognition, treatment, and control of hypertension in the Danish population-based Lolland-Falster Health Study

**DOI:** 10.1093/eurpub/ckag117

**Published:** 2026-07-09

**Authors:** Ebba Mannheimer, Kristine Hommel, Morten Buus Jørgensen, Anne-Lise Kamper, Randi Jepsen, Bo Feldt-Rasmussen, Mads Hornum

**Affiliations:** Department of Nephrology and Endocrinology, Rigshospitalet, Copenhagen, Denmark; Department of Medicine, Holbæk Hospital, Holbæk, Denmark; Department of Clinical Medicine, Faculty of Health and Medical Sciences, University of Copenhagen, Copenhagen, Denmark; Department of Nephrology and Endocrinology, Rigshospitalet, Copenhagen, Denmark; Department of Clinical Medicine, Faculty of Health and Medical Sciences, University of Copenhagen, Copenhagen, Denmark; Department of Nephrology and Endocrinology, Rigshospitalet, Copenhagen, Denmark; Lolland-Falster Health Study, Centre for Health Research, Zealand University Hospital, Nykøbing F, Denmark; Department of Nephrology and Endocrinology, Rigshospitalet, Copenhagen, Denmark; Department of Clinical Medicine, Faculty of Health and Medical Sciences, University of Copenhagen, Copenhagen, Denmark; Department of Nephrology and Endocrinology, Rigshospitalet, Copenhagen, Denmark; Department of Clinical Medicine, Faculty of Health and Medical Sciences, University of Copenhagen, Copenhagen, Denmark

## Abstract

In the Danish population-based Lolland-Falster Health Study (LOFUS), conducted in a socioeconomically disadvantaged area with marked regional health inequalities, we recently identified a hypertension prevalence of 45%. A more detailed understanding of recognition, control, and treatment of hypertension in this setting may help improve interventions to reduce hypertension-related cardiovascular disease. This was a cross-sectional study including participants with hypertension identified in LOFUS using data from clinical examinations and questionnaires. Recognition was assessed by a registered diagnosis or self-reported hypertension and factors associated with recognition status were analyzed. Cardiovascular risk stratification was applied to individuals with unrecognized hypertension to determine treatment indication. Blood pressure (BP) control and antihypertensive treatment patterns were evaluated among participants with recognized hypertension. Among 7329 participants with hypertension, 39% were unrecognized. Individuals with unrecognized hypertension were younger, had higher educational attainment and fewer comorbidities than those with recognized hypertension. Larger household size was associated with higher odds of unrecognition. Two-thirds of individuals with unrecognized hypertension were classified as having high 10-year cardiovascular risk and met criteria for antihypertensive treatment. Among participants with recognized hypertension, 51% had uncontrolled BP despite >50% receiving ≥2 antihypertensive agents. The group with uncontrolled BP had fewer comorbidities than those with controlled BP. In this rural cohort with a high prevalence of hypertension, a large proportion remained unrecognized despite elevated cardiovascular risk, and among individuals with recognized hypertension, treatment targets were often not achieved. These findings indicate a substantial unmet need for improved recognition and management of hypertension, with important implications for public health.

## Introduction

Globally, hypertension affects approximately one in three adults aged 30–79 years [1] and is the most important modifiable risk factor for cardiovascular morbidity and mortality [2]. Both systolic and diastolic blood pressure (BP) levels are strongly associated with the risk of cardiovascular disease (CVD) including stroke and coronary heart disease [3]. In addition to BP levels, the accumulation of other cardiovascular risk factors such as dyslipidaemia, obesity, diabetes, and chronic kidney disease (CKD) further contributes to the development of CVD in individuals with hypertension. Conversely, hypertension is a major contributor to the development and progression of CKD. Effective management of BP, alongside recognition and treatment of the overall cardiovascular risk profile, can substantially reduce the risk of CVD and CVD-related death [4]. However, despite effective low-cost treatment options, many individuals with hypertension remain undiagnosed or without treatment [5].

In Denmark, studies have reported a hypertension prevalence ranging from 25% to 38.5% with less than 50% aware of the diagnosis and even fewer receiving treatment [6–8]. The Danish population-based Lolland-Falster Health Study (LOFUS) [9] with over 16 000 adult participants—was conducted in a rural-provincial area characterized by significantly shorter life expectancy and lower socioeconomic status compared to the national average. In this cohort, we previously reported a hypertension prevalence of 45%, with about half of affected individuals unaware of their condition [10]. Additionally, 70% of individuals with hypertension had elevated BP (≥140/90 mmHg) at the study examination, indicating a substantial burden of uncontrolled or untreated hypertension and a potentially important, modifiable contributor to the adverse health outcomes in Lolland-Falster [10].

Building on these findings, this cross-sectional study aimed to assess recognition, BP control, and treatment of hypertension in LOFUS. Among individuals with recognized hypertension, we evaluated BP control and antihypertensive treatment patterns. For those with unrecognized hypertension, we examined demographic and clinical characteristics associated with lack of recognition and determined the proportion meeting indications for antihypertensive treatment based on cardiovascular risk stratification. We hypothesized that fewer than 50% of treated individuals achieve BP control and that more than 50% of individuals with unrecognized hypertension meet criteria for antihypertensive treatment.

## Methods

### Setting and data sources

This cross-sectional study is based on data from the household- and population-based LOFUS study, conducted in Lolland-Falster, a mixed-rural provincial area of Denmark with around 105 000 inhabitants. The overall aim of LOFUS was to establish baseline knowledge about factors that influence health in this area. Individuals were randomly selected and invited to participate together with their household members. Between 2016 and 2020, a total of 16 142 adult individuals (participation rate 36%) [11] attended one of the three study sites and underwent a physical examination and provided blood and urine samples [9]. Prior to the study visit, all individuals completed an electronic questionnaire on their health status, medication use, and socioeconomic factors. Further details about LOFUS are available in the published study protocol [9].

Denmark has a tax-funded health-care system which is freely accessible to all citizens. By law, all hospital contacts and dispensed prescriptions are recorded in nationwide health registers, including the Danish National Patient Register (DNPR), which contains information on all inpatient, outpatient, and emergency room contacts at public and private hospitals [12]. Each contact is assigned at least one diagnosis based on the International Classification of Diseases, 10th Revision (ICD-10). The Danish Register of Pharmaceutical Sales (DRPS) holds information on all prescriptions dispensed at community pharmacies in Denmark [13]. All Danish citizens are assigned a unique Civil Personal Register number at birth or immigration, which enabled individual-level linkage of LOFUS data to the health registers.

### Study sample

Adult participants (≥18 years) with preexisting diagnosis of hypertension or hypertension identified in LOFUS were included in the current study, excluding those without BP measurements. Hypertension was defined by one or more of the following criteria [1, 14]:

- systolic BP (SBP) ≥140 mmHg,
- diastolic BP (DBP) ≥90 mmHg,
- self-reported diagnosis of hypertension,
- self-reported use of antihypertensive medication, or
- a recorded hypertension diagnosis in the DNPR (ICD-10 codes listed in Table S1).

### BP measurements

BP was measured by trained healthcare professionals with an electronic BP device (Connex ProBP 3400, Welch Allyn, USA) according to a standardized protocol. Measurements were obtained from the upper left arm using an appropriately sized cuff; if measurement on the left arm was contraindicated, the right arm was used instead. During the measurements, participants were instructed to avoid moving or speaking and were positioned supine. The last of three consecutive BP measurements was used for analysis; if the last measurement was missing, the second was used, and if both were missing, the first was used.

### Study outcomes

#### Recognition of hypertension

Recognized hypertension included those who self-reported hypertension, self-reported use of antihypertensive medication, or had a recorded hypertension diagnosis in the DNPR, regardless of study BP. Unrecognized hypertension was defined as SBP ≥140 mmHg or DBP ≥90 mmHg in individuals who did not report hypertension, did not report use of antihypertensive medication, and had no recorded hypertension diagnosis in the DNPR.

#### BP control and antihypertensive treatment in recognized hypertension

Controlled BP was defined as BP <140/90 mmHg in accordance with existing guidelines during the study period [14, 15]. The use of antihypertensive treatment was defined as having a redeemed prescription in the DRPS for any of the following BP-lowering drugs: renin-angiotensin system (RAS) inhibitors, calcium channel blockers, diuretics, beta-blockers, or antiadrenergic agents within 12 months preceding the study visit.

#### Indication for antihypertensive treatment among individuals with unrecognized hypertension

Indication for antihypertensive treatment was determined using the cardiovascular risk stratification and treatment algorithm shown in Table S2, which is based on the Danish national hypertension guidelines applicable at the time of the study [14] and supplemented by the European guidelines [15, 16]. Risk stratification incorporated cardiovascular risk factors and asymptomatic organ damage, as defined in Table S3. Participants were categorized as having low (<15%), moderate (15%–20%), high (20%–30%), or very high (>30%) 10-year risk of cardiovascular morbidity and mortality.

### Definitions of clinical and sociodemographic variables

The definition of CKD was based on an estimated glomerular filtration rate (eGFR) <60 ml/min/1.73 m^2^, a urine albumin-to-creatinine ratio (UACR) ≥30 mg/g, or a registered CKD diagnosis in the DNPR. CVD was defined as a history of atrial fibrillation, ischemic heart disease, heart failure, peripheral vascular disease, or stroke, either self-reported or registered in the DNPR. Diabetes was defined as HbA1c ≥48 mmol/mol, self-reported diabetes, self-reported use of glucose-lowering medication, or a registered diagnosis of diabetes. ICD-10 codes used for identifying CKD, CVD, and diabetes are listed in Table S1. Abdominal obesity was defined as waist circumference of ≥88 cm for women and ≥102 cm for men. Dyslipidaemia was defined as total cholesterol >4.9 mmol/L, LDL cholesterol >3.0 mmol/L, HDL cholesterol <1.0 mmol/L in men or <1.2 mmol/L in women, or triglycerides >1.7 mmol/L [14].

Socioeconomic status was assessed using self-reported educational level and occupational status. The specific response categories and their classification are presented in Table S4.

### Statistics

Continuous variables are reported as means with standard deviations or medians with interquartile ranges and compared using the two-sample *t*-test or the Wilcoxon rank-sum test. Age groups were defined based on completed years and categorized into four strata: 18–55, 56–65, 66–75, and ≥76 years. Categorical variables are presented as proportions and compared using the chi-squared test. Associations with hypertension recognition status were examined using univariable logistic regression, reporting odds ratios (ORs) with 95% confidence intervals (CIs). These analyses were intended to describe patterns of association between individual characteristics and hypertension recognition status rather than identify independent predictors. For analyses not involving household-derived exposures, generalized linear mixed-effects models with household included as a random effect was applied to account for clustering, as approximately 70% of participants were enrolled together with one or more adult household members. A *P-*value<.05 was considered statistically significant.

### Ethics approval and consent to participate

All participants provided written informed consent in compliance with the Declaration of Helsinki, covering, but not limited to, storage of biological samples and linkage to health registers. LOFUS has been approved by the Region of Zealand’s Ethical Committee on Health Research (SJ-421) and is registered with ClinicalTrials.gov (NCT02482896; registration date: June 26, 2015). Both LOFUS and the current study are registered with the Danish Data Protection Agency (P-2024-16360 and P-788-2022).

## Results

### Participant characteristics and recognition status

After excluding five individuals without BP measurements, the study sample included 7329 participants with hypertension, corresponding to 45% of the adult LOFUS cohort. Among participants with hypertension, 2859 (39%) had unrecognized hypertension, while 4470 (61%) had recognized hypertension. Among those with recognized hypertension, 15% had a registered hospital diagnosis and 98% self-reported hypertension and/or use of antihypertensive medication. Among participants with a registered diagnosis, 87% also self-reported hypertension. Participant demographics and clinical characteristics are presented in Table 1, stratified by hypertension recognition status. Individuals with unrecognized hypertension were significantly younger than those with recognized hypertension (median age 63 vs. 68 years; *P *< .001). Occupational activity differed between groups, with 48% of individuals with unrecognized hypertension being occupationally active compared with 31% of those with recognized hypertension. Most individuals had a short postsecondary education level and reported not smoking, with no differences between the groups. Households with more than three members were more common in the unrecognized group. Moreover, 17% in the unrecognized group lived with at least one other household member with unrecognized hypertension, compared to 14% in the recognized group (*P *< .001).

**Table 1.** Demographic and clinical characteristics of the participants with hypertension according to recognition status.

	Total (*N* = 7329)	Unrecognized (*n *= 2859)	Recognized (*n *= 4470)
Sex—*n* (%)			
Male	3769 (51.4)	1505 (52.6)	2264 (50.6)
Female	3560 (48.6)	1354 (47.4)	2206 (49.4)
Age (years), median (IQR)	66.3 (57.9–72.8)	63.3 (54.4–70.8)	67.9 (60.3–73.9)
Age distribution (years)—*n* (%)			
18–55	1529 (20.9)	823 (28.8)	706 (15.8)
56–65	2052 (28.0)	854 (29.9)	1198 (26.8)
66–75	2625 (35.8)	870 (30.4)	1755 (39.3)
≥76	1123 (15.3)	312 (10.9)	811 (18.1)
Systolic blood pressure (mmHg), mean (SD)	146 (17.8)	154 (12.1)	142 (19.2)
Diastolic blood pressure (mmHg), mean (SD)	83.3 (9.14)	86.6 (8.65)	81.2 (8.83)
Blood pressure levels (mmHg)—*n* (%)			
<130/<80	1219 (16.6)	0 (0)	1219 (27.3)
130–139/85–89	951 (13.0)	0 (0)	951 (21.3)
140–159/90–99	3578 (48.8)	2064 (72.2)	1514 (33.9)
160–179/100–109	1282 (17.5)	653 (22.8)	629 (14.1)
≥180/≥110	299 (4.1)	142 (5.0)	157 (3.5)
Educational level, self-reported—*n* (%)			
Medium/long postsecondary education	1543 (22.0)	623 (23.5)	920 (21.0)
Short postsecondary education	4164 (59.3)	1608 (60.6)	2556 (58.4)
No education beyond secondary school	1320 (18.8)	423 (15.9)	897 (20.5)
Occupational status, self-reported—*n* (%)			
Active	2687 (37.8)	1294 (48.5)	1393 (31.4)
Temporarily not active	177 (2.5)	57 (2.1)	120 (2.7)
Inactive	4092 (57.6)	1269 (47.5)	2823 (63.7)
Other	145 (2.0)	50 (1.9)	95 (2.1)
Household size—*n* (%)			
1 (alone)	1356 (18.5)	460 (16.1)	896 (20.0)
2	4678 (63.8)	1729 (60.5)	2949 (66.0)
3–4	1122 (15.3)	566 (19.8)	556 (12.4)
≥5	173 (2.4)	104 (3.6)	69 (1.5)
Living with ≥1 other household member with hypertension (excluding oneself)—*n* (%)	2955 (40.3)	1088 (38.1)	1867 (41.8)
Living with ≥1 other household member with unrecognized hypertension (excluding oneself)—*n* (%)	1096 (15.0)	486 (17.0)	610 (13.6)

IQR, interquartile range; SD, standard deviation.

Diabetes and CVD were three times more common among individuals with recognized hypertension (Table 1). Abdominal obesity and CKD were also more common among those with recognized hypertension, including the more severe stages of CKD.

Figure S1 shows mean BP by age and recognition status. Across all age groups, individuals with unrecognized hypertension had higher SBP than those with recognized hypertension. SBP increased with age in both groups, whereas DBP declined with age. More than one in four (28%) with unrecognized hypertension had BP ≥160/100 mmHg, compared to 17% in the recognized group, while BP ≥180/110 mmHg was uncommon in both groups (<5%).

### Factors associated with having unrecognized hypertension

Increasing age was associated with lower odds of unrecognized hypertension compared with individuals aged 18–55 years (Table 2). No significant association with sex was observed. Individuals without education beyond secondary school and those who were not occupationally active had lower odds of unrecognized hypertension. In contrast, larger household size was associated with significantly higher odds. Living with another person with hypertension, regardless of recognition status, was associated with lower odds of unrecognized hypertension; however, living with another person with unrecognized hypertension was associated with higher odds. Concomitant CVD, diabetes, CKD, and abdominal obesity were all associated with lower odds of unrecognized hypertension.

**Table 2.** Factors associated with having unrecognized hypertension.

		Univariate analysis; OR (95% CI)
Female sex		0.92 (0.83–1.02)
Age group	18–55 years	Reference
	56–65 years	0.58 (0.5–0.68)
	66–75 years	0.39 (0.33–0.45)
	≥76 years	0.30 (0.24–0.36)
Smoking		1.13 (0.98–1.31)
Educational level	Medium/long postsecondary education	Reference
	Short postsecondary education	0.93 (0.81–1.06)
	No education beyond secondary school	0.68 (0.57–0.81)
Occupational status	Active	Reference
	Temporarily not active	0.48 (0.34–0.69)
	Inactive	0.46 (0.41–0.51)
	Other	0.54 (0.37–0.80)
Household size	1 (alone)	Reference
	2	1.17 (1.01–1.35)
	3–4	2.17 (1.79–2.62)
	≥5	3.37 (2.32–4.91)
Living with ≥1 other household member with hypertension (excluding oneself)		0.86 (0.78–0.94)
Living with ≥1 other household member with unrecognized hypertension (excluding oneself)		1.30 (1.14–1.48)
Abdominal obesity		0.48 (0.42–0.54)
Cardiovascular disease		0.18 (0.15–0.22)
Chronic kidney disease		0.55 (0.49–0.63)
Diabetes		0.21 (0.17–0.27)

CI, confidence interval; OR, odds ratio.

### Indication for antihypertensive treatment in individuals with unrecognized hypertension

**Figure 1.**
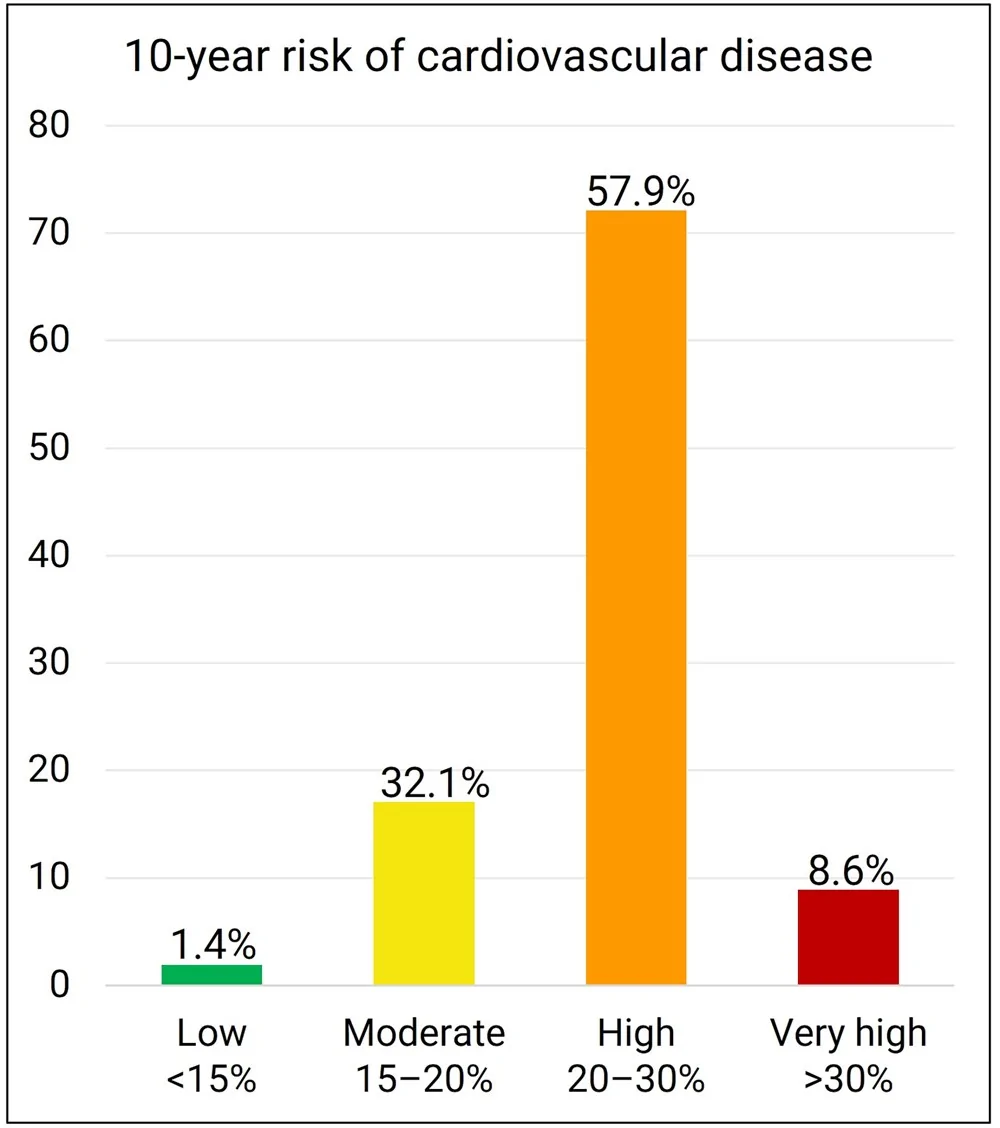
Category of absolute 10-year risk of cardiovascular disease among individuals with unrecognized hypertension (*n* = 2859).

Among individuals with unrecognized hypertension, the most common additional CVD risk factor was dyslipidaemia (>82%) followed by age, male sex, and abdominal obesity (Table S5). Overall, 60% had three or more concomitant risk factors. More than half showed evidence of asymptomatic organ damage (eGFR 30–60 ml/min/1.73 m^2^, albuminuria, or high pulse pressure) whereas established CVD or severe CKD was present in fewer than one in ten. The majority (58%) were classified as having a high estimated 10-year risk of CVD, while an additional 9% were classified as very high risk. By comparison, 32% had a moderate estimated 10-year risk, and only 1% had a low risk (Figure 1; Table S2). Based on this risk stratification, two-thirds of individuals with unrecognized hypertension met criteria for antihypertensive treatment in addition to lifestyle modification, while the remaining one-third met criteria for lifestyle modification alone as initial management, with pharmacological treatment considered if BP targets were not subsequently achieved.

### BP control of individuals with recognized hypertension

Among individuals with recognized hypertension, 2170 (49%) had controlled BP, while the remaining had uncontrolled BP (Table 3). When applying the current guideline-recommended threshold of <130/80 mmHg [17], only 27% had BP below target (Table 1). BP control was more common among women than men. Age was slightly lower, and comorbidities were generally more prevalent in the controlled group, except for CKD. Among individuals with uncontrolled hypertension, 11% had no recorded antihypertensive treatment and 37% were receiving monotherapy, compared with 6% and 33% in the controlled group, respectively. Use of multiple (≥2) agents was more frequent in individuals with controlled hypertension (61% vs. 53%; *P *< .001). In decreasing order, the most commonly prescribed agents were RAS inhibitors (>60%), diuretics, calcium channel blockers, and beta-blockers in both groups. Among participants who self-reported using antihypertensive medication, 98% had at least one redeemed prescription within 12 months before their study visit.

**Table 3.** Characteristics of individuals with recognized hypertension according to blood pressure control.

	Uncontrolled blood pressure ≥140/90 mmHg (*n *= 2300)	Controlled blood pressure <140/90 mmHg (*n *= 2170)
Female sex—*n* (%)	1070 (46.5)	1136 (52.4)
Age (years), median (IQR)	68.6 (61.3–74.3)	67.1 (59.2–73.4)
Registered hypertension diagnosis—*n* (%)	368 (16.0)	286 (13.2)
Cardiovascular disease—*n* (%)	551 (24.0)	661 (30.5)
Atrial fibrillation—*n* (%)	137 (6.0)	180 (8.3)
Ischemic heart disease—*n* (%)	346 (15.0)	468 (21.6)
Heart failure—*n* (%)	25 (1.1)	89 (4.1)
Stroke—*n* (%)	77 (3.3)	70 (3.2)
Peripheral vascular disease—*n* (%)	88 (3.8)	76 (3.5)
Chronic kidney disease—*n* (%)	797 (34.7)	608 (28.0)
Diabetes—*n* (%)	347 (15.1)	382 (17.6)
Abdominal obesity—*n* (%)	1457 (63.3)	1517 (69.9)
Antihypertensive medication—*n* (%)		
Renin-angiotensin system inhibitors	1398 (60.8)	1415 (65.2)
Calcium channel blockers	880 (38.3)	837 (38.6)
Diuretics	917 (39.9)	1056 (48.7)
Beta-blocking agents	664 (28.9)	733 (33.8)
Antiadrenergic agents	32 (1.4)	25 (1.2)
Number of antihypertensives—*n* (%)		
None recorded	245 (10.7)	128 (5.9)
1	844 (36.7)	717 (33.0)
2	715 (31.1)	756 (34.8)
≥3	496 (21.5)	569 (26.3)

IQR, interquartile range.

## Discussion

In this population-based cohort, 39% of individuals with hypertension remained unrecognized and were significantly younger, had fewer comorbidities, and higher educational attainment than those with recognized hypertension. Nevertheless, a substantial proportion had a high estimated 10-year risk of CVD and met guideline-based criteria for antihypertensive treatment. More than half of those with recognized hypertension had uncontrolled BP, including many receiving combination therapy.

### Prevalence and recognition of hypertension

The hypertension prevalence observed in LOFUS of 45% [10] was higher than that reported in previous national estimates but in line with estimates from the United States [18] and other European countries [19]. World Health Organization (WHO) modeling based on 2019 data indicates an age-standardized prevalence of approximately 36% in Denmark [20], while earlier Danish surveys reported 22% using home BP confirmation [7] and 38.5% using office measurements [6], with both surveys also incorporating self-reported hypertension. The higher prevalence in LOFUS may partly reflect differences in study design and participant characteristics, including the high prevalence of cardiometabolic risk factors such as obesity (26%) and CKD (18%), previously reported in this cohort [10]. In comparison, the prevalence of obesity in the general Danish population was estimated at 17% at the time of the study [21].

These findings, nonetheless, indicate substantial hypertension burden in rural Denmark. In LOFUS, SBP increased with age while DBP declined, reflecting well-established age-related arterial stiffening [22] and suggesting that the ongoing population aging will further amplify the burden of systolic hypertension. According to WHO, over half of CVD deaths in Denmark in 2019 were attributed to high SBP and under an improved hypertension control scenario, up to 15 000 deaths could be averted by 2040 [20].

At the same time, 40% of individuals with hypertension in LOFUS remained unrecognized—were unaware of their condition and without a registered diagnosis. This proportion is similar to global estimates (44%) [1] and findings from other high-income countries [23, 24]. In contrast, previous data from Denmark have varied widely from 28–60%, with the highest proportions of unawareness in studies based on office BP measurements without confirmation by home-BP monitoring [6–8, 20].

Among participants with a registered hypertension diagnosis, 87% also self-reported hypertension, indicating high awareness among individuals with physician-recognized disease. This proportion stands in contrast to that previously reported for CKD in this cohort, where less than 40% of individuals with a registered CKD diagnosis self-reported kidney disease [10]. Given that both hypertension and CKD are asymptomatic in most patients, this difference in awareness is particularly noteworthy. The difference may reflect the more explicit disease-specific treatment of hypertension, with clearly labeled BP-lowering medications, whereas CKD management largely relies on therapies that may not be perceived as directly targeting kidney disease. In addition, BP can be easily monitored at home, which may further facilitate patient understanding and engagement, in contrast to kidney function, which is less tangible to patients. Given the close bidirectional relationship between hypertension and CKD, improving awareness and management of both conditions is important for effective cardiovascular and renal risk reduction.

### Characteristics, cardiovascular risk, and treatment eligibility of unrecognized hypertension

Consistent with existing research [6, 25, 26], the profile of individuals with unrecognized hypertension in LOFUS—on average younger, higher educational attainment, more often occupationally active, with fewer comorbidities—suggests that contact with the healthcare system is an important determinant of detection. A British study proposed that higher rates of hypertension underdiagnosis among women with higher educational attainment may partly relate to differences in use of reproductive healthcare, as women with higher educational attainment tend to have fewer children on average [26].

In addition, living with another person with hypertension, regardless of recognition status, was associated with lower odds of unrecognized hypertension in LOFUS, possibly reflecting increased awareness and attention to BP within the household. At the same time, the clustering of unrecognized hypertension within households suggests shared healthcare-seeking behavior, potentially influenced by access to the same primary care provider.

These findings support the potential value of household- or community-based screening to reach individuals with limited healthcare contact. In the United States, screening initiatives in nontraditional settings such as barbershops [27] and laundromats [28] have successfully identified individuals with previously undiagnosed hypertension. However, evidence on the effectiveness of population-wide hypertension screening for reducing cardiovascular morbidity and mortality remains conflicting [2]. Previous Danish randomized controlled cardiovascular screening trials, including the Viborg Vascular (VIVA) trial [29] and the Danish Cardiovascular Screening (DANCAVAS) trial [30], enrolled more than 50 000 and 46 000 men aged 65–74 years, respectively. Screening was associated with a significant reduction in all-cause mortality in the VIVA trial [29], whereas in DANCAVAS it increased the detection of unrecognized conditions, including hypertension, without a significant reduction in all-cause mortality [30]. Nonetheless, further evaluation of targeted detection strategies may be warranted in high-burden rural–provincial settings similar to Lolland-Falster.

Paradoxically, these apparently healthier individuals with unrecognized hypertension faced considerable cardiovascular risk. In LOFUS, nearly two-thirds of participants with unrecognized hypertension were classified as having a high or very high estimated 10-year risk of CVD and met criteria for antihypertensive treatment in addition to lifestyle modification. This proportion is in line with findings from a previous Danish population-based study (*n *= 6784) [8] and a Swiss primary care study (*n *= 1003), where the majority of patients with hypertension were classified as high/very high risk, and dyslipidaemia and abdominal obesity were the most common modifiable risk factors, mirroring our observations [31]. Notably, the introduction of SCORE2/SCORE2-OP for quantitative cardiovascular risk assessment in the recent European guidelines [2, 14], tends to classify a larger proportion of individuals as high risk [32], suggesting that contemporary application would not weaken the present findings.

### BP control and treatment patterns in recognized hypertension

Lack of BP control is a well-known challenge in hypertension, with control rates varying widely across countries [6, 24, 33]. Among LOFUS participants with recognized hypertension half had controlled BP which is similar to a previous Danish study from 2015 where 51% of treated individuals achieved BP below target [8]. In contrast, in a large Danish primary care study from 2012 only 33% had controlled BP [34] and in studies from Switzerland [24] and the UK control rates were 42% and 38%, respectively [25]. The more favorable findings in LOFUS may partly reflect improving temporal trends in BP control reported in Denmark [35] and other high-income countries [23]. In addition, since LOFUS was conducted, contemporary guidelines have placed greater emphasis on lower BP targets (<130/80 mmHg) and earlier initiation of combination therapy, preferably as single-pill combinations to simplify treatment regimens and improve adherence [17, 36]. When applying the stricter <130/80 mmHg threshold, only 27% achieved controlled BP, underscoring a clear need for enhanced hypertension management in Lolland-Falster.

Lower use of combination therapy (≥2 drugs) in the uncontrolled group suggests insufficient treatment intensification; however, as more than half were already receiving combination therapy, this alone is unlikely to explain poor BP control. Instead, differences in comorbidity burden may reflect variations in healthcare contact. In line with other studies [25, 34], comorbidities were generally more prevalent in the controlled group, likely resulting in more frequent clinical encounters and greater opportunities for treatment adjustment and lifestyle counseling. The exception for CKD is clinically plausible, given the complexity of BP management in CKD and the potential for more resistant hypertension or competing treatment constraints (e.g. tolerability, polypharmacy).

Beyond individual-level clinical factors, healthcare system capacity is also likely to influence BP management. Despite Denmark’s universal, free-access healthcare system, regional disparities in healthcare provision persist. The region, which includes Lolland-Falster faces challenges in recruiting general practitioners and other health professionals and has the lowest density of general practitioners per 1000 inhabitants in Denmark [37]. This limited workforce capacity likely constrains access to care and continuity and might contribute to suboptimal BP recognition and control. The persistence of substantial gaps in hypertension recognition, treatment, and control despite universal healthcare access and the widespread availability of effective antihypertensive treatment highlights that implementation of hypertension care remains challenging even in well-resourced healthcare systems.

### Strengths and limitations

Some limitations should be considered when interpreting the findings of this study. As a consequence of the cross-sectional design, BP was assessed at a single study visit. Clinical guidelines recommend confirmatory assessment using ambulatory or home BP monitoring in routine clinical practice to avoid overlooking masked hypertension and overtreating white-coat hypertension [38] or well-controlled hypertension [2]. Although BP was based on three consecutive readings using a standardized protocol, there is a risk of misclassification which may have led to overestimation of the prevalence of hypertension, including the proportion with uncontrolled hypertension [39]. The same applies to blood and urine samples, which formed the basis of defining CVD risk factors such as CKD and diabetes. However, out-of-office confirmation and repeated measurements are rarely feasible in large observational studies such as LOFUS.

The use of questionnaire data to assess hypertension recognition may have been subject to recall or reporting bias. Yet, 98% of individuals who reported using antihypertensive medication had at least one fulfilled prescription recorded in national registers, indicating good concordance between self-reported and register-based data. Moreover, although the Danish prescription registers provide near-complete population coverage, some antihypertensive agents are prescribed for conditions other than hypertension, such as heart failure, arrhythmias, or benign prostatic hyperplasia, which may have slightly overestimated treatment rates. However, these medications have BP–lowering effects and thus remain clinically relevant in this context.

Participation bias represents an important limitation, as only 36% of invited individuals participated in LOFUS [40]. In a register-based follow-up study, Holmager *et al.* [11] observed that nonparticipation in LOFUS was more common among individuals with lower socioeconomic status, including those receiving public support rather than being self-supporting, as well as among men, younger adults, and in-migrants rather than long-term residents. In the years following invitation, non-participants also had approximately three-fold higher mortality than participants [11]. This indicates that the study sample is likely healthier than the source population, and the true burden of hypertension in Lolland-Falster may be higher than that reported here. Furthermore, the household-based design may have introduced clustering of hypertension and related behaviors within households, which could affect the precision of prevalence estimates and should be considered when interpreting these findings.

Finally, as the study is conducted in a rural–provincial area, the findings are not directly generalizable to urban populations. However, this specific context also represents a key strength. To our knowledge, this is among the largest European studies to assess recognition, treatment, and control of hypertension in a rural–provincial area characterized by lower socioeconomic status, lower physician density [37], and shorter life expectancy compared with urban areas [9]. Moreover, the unique household-based design of LOFUS enables assessment of household structure and clustering in relation to unrecognized hypertension.

## Conclusion

In this large population-based study from rural Denmark, nearly 40% of participants with hypertension remained unrecognized despite high cardiovascular risk and more than half of individuals with recognized hypertension did not achieve BP control. These findings highlight the potential to improve cardiovascular health through better detection and management of hypertension, thereby narrowing regional health inequalities in Lolland-Falster and in similar settings.

## Supplementary Material

ckag117_Supplementary_Data

## Data Availability

Data from third parties are not publicly available but can be requested from LOFUS, Statistics Denmark, and the Danish Health Data Authority, in compliance with Danish legislation. Key points In a large Danish population-based study conducted in a socioeconomically disadvantaged area with marked regional health inequalities, hypertension affected 45% of participants. A more detailed understanding of recognition, control, and treatment of hypertension in this setting provides insight into opportunities for reducing hypertension-related cardiovascular disease and health inequalities. Nearly 40% of individuals with hypertension were unrecognized; the majority of these were at high cardiovascular risk and met guideline-based criteria for antihypertensive treatment. Over half of individuals with recognized hypertension did not meet blood pressure targets. These findings indicate a substantial unmet need for improved recognition and management of hypertension, with important implications for public health.

## Supplementary data

Conflict of interest: MH has received honoraria for participation in advisory boards from AstraZeneca, Bayer, Boehringer Ingelheim, Vifor, GSK, and Novo Nordisk A/S, as well as speaker fees from AstraZeneca, Boehringer Ingelheim, and Novo Nordisk A/S, all unrelated to the present work. EM has received research funding from the Danish Kidney Association, the Danish Society of Nephrology, and the Helen and Ejnar Bjørnow Foundation. All other authors declare no competing interests.
