# Supplementary material for: Recognition, treatment, and control of hypertension in the Danish population-based Lolland-Falster Health Study
Source: Eur J Public Health. 2026 Jul 9;36(4):ckag117. doi: 10.1093/eurpub/ckag117 (PMC13349664; doi:10.1093/eurpub/ckag117)
Supplement: ckag117_Supplementary_Data [file ckag117_supplementary_data.zip › ejph-2026-02-om-0197-File008.docx]

| **Table S4. Categorization of socioeconomic data** |
| --- |
| Data on socioeconomic status were obtained from the questionnaire. The question regarding occupational status had 16 different response options. However, these were divided into the following four categories in the analyses: |
| ***Active:*** employee; self-employed; combined employee and self-employed; in the military; secondary school pupil; postsecondary student; apprentice; assisting spouse; housewife/househusband |
| ***Temporarily inactive:*** unemployed; undergoing rehabilitation; on sickness leave for 3 months or more |
| ***Inactive****:* retired due to age; recipient of disability benefit; early retirement |
| ***Other****:* other |
|  |
| Seven different response options for educational level were divided into the following three categories for analyses: |
| ***No Postsecondary education*** |
| ***Short Postsecondary education****:* unspecified other education; one or more short courses; vocational education; short higher education for 2–3 years |
| ***Medium or long postsecondary education***: medium higher education for 3–4 years; long higher education for > 4 years |
