# Supplementary material for: Recognition, treatment, and control of hypertension in the Danish population-based Lolland-Falster Health Study
Source: Eur J Public Health. 2026 Jul 9;36(4):ckag117. doi: 10.1093/eurpub/ckag117 (PMC13349664; doi:10.1093/eurpub/ckag117)
Supplement: ckag117_Supplementary_Data [file ckag117_supplementary_data.zip › ejph-2026-02-om-0197-File006.docx]

| **Table S2. Treatment recommendations according to blood pressure level and cardiovascular risk** | | | |
| --- | --- | --- | --- |
| **Risk factors, asymptomatic organ damage or disease** | **BP levels (mmHg)** | | |
|  | **Systolic BP 140–159 or diastolic BP 90–99** | **Systolic BP 160–179 or diastolic BP 100–109** | **Systolic BP ≥ 180 or diastolic BP ≥ 110** |
| **No risk factors** | Lifestyle changes ± antihypertensive treatment if BP target not achieved | Lifestyle changes ± antihypertensive treatment if BP target not achieved | Lifestyle changes + immediate antihypertensive treatment |
| **1–2 risk factors** | Lifestyle changes ± antihypertensive treatment if BP target not achieved | Lifestyle changes ± antihypertensive treatment if BP target not achieved | Lifestyle changes + immediate antihypertensive treatment |
| **≥ 3 risk factors** | Lifestyle changes ± antihypertensive treatment if BP target not achieved | Lifestyle changes + antihypertensive treatment | Lifestyle changes + immediate antihypertensive treatment |
| **Asymptomatic organ damage, CKD stage 3, diabetes mellitus** | Lifestyle changes + antihypertensive treatment | Lifestyle changes + antihypertensive treatment | Lifestyle change + immediate antihypertensive treatment |
| **Symptomatic CVD, CKD stages 4–5, diabetes with organ damage** | Lifestyle changes + antihypertensive treatment | Lifestyle changes + antihypertensive treatment | Lifestyle changes + immediate antihypertensive treatment |
| **Absolute 10-year risk of stroke or myocardial infarction: low (< 15%, green), moderate (15–20%, yellow), high (20–30%, orange), and very high (> 30%, red), with corresponding treatment consequences to be drawn from the stratification. Definitions of CVD, CKD, cardiovascular risk factors, and asymptomatic organ damage are provided in Table S3. Figure adapted from the Danish Hypertensio Arterialis - Treatment Guideline 2015.**  **BP: blood pressure; CKD, chronic kidney disease; CVD, cardiovascular disease.** | | | |
