# Supplementary material for: Recognition, treatment, and control of hypertension in the Danish population-based Lolland-Falster Health Study
Source: Eur J Public Health. 2026 Jul 9;36(4):ckag117. doi: 10.1093/eurpub/ckag117 (PMC13349664; doi:10.1093/eurpub/ckag117)
Supplement: ckag117_Supplementary_Data [file ckag117_supplementary_data.zip › ejph-2026-02-om-0197-File009.docx]

| **Table S5. Prevalence of cardiovascular risk factors, asymptomatic organ damage, diabetes, established cardiovascular disease, and severe chronic kidney disease among individuals with unrecognized hypertension, used for cardiovascular risk stratification and assessment of guideline-directed treatment indication.** | |
| --- | --- |
| **Parameters** | **n (%)** |
| **Cardiovascular risk factors** |  |
| Male sex | 1,505 (52.6) |
| Age [male: 55 years, female: 65 years] | 1,612 (56.4) |
| BMI > 30 kg/m^2^ | 713 (25.1) |
| Abdominal obesity | 1,454 (50.9) |
| Dyslipidemia | 2,351 (82.7) |
| Smoking | 441 (16.7) |
| **Total number of risk factors** | ≥ 3: 1,714 (59.9) · 1–2: 1,074 (37.6) · None: 71 (2.5) |
|  |  |
| **Diabetes or signs of organ damage** |  |
| Diabetes | 130 (4.5) |
| Chronic kidney disease stage 3 | 110 (3.8) |
| Urine albumin-creatinine ratio 30–299 mg/g | 496 (17.9) |
| Pulse pressure > 60 mmHg & age > 60 years | 1,494 (52.3) |
| **In total** | 1,562 (54.6) |
|  |  |
| **Established cardiovascular disease, severe chronic kidney disease or diabetes with complications** |  |
| Cardiovascular disease | 214 (7.5) |
| Chronic kidney disease stages 4–5 and/or urine  albumin-creatinine ratio > 300 mg/g | 38 (6.3) |
| Diabetes with complications | 13 (0.5) |
| **In total** | 245 (8.6) |
