# Supplementary material for: Recognition, treatment, and control of hypertension in the Danish population-based Lolland-Falster Health Study
Source: Eur J Public Health. 2026 Jul 9;36(4):ckag117. doi: 10.1093/eurpub/ckag117 (PMC13349664; doi:10.1093/eurpub/ckag117)
Supplement: ckag117_Supplementary_Data [file ckag117_supplementary_data.zip › ejph-2026-02-om-0197-File005.docx]

**Table S1. ICD-10 codes and respective diagnoses used to identify individuals with hypertension and other comorbidities**

**Hypertensive diseases**

| I10 | Essential (primary) hypertension |
| --- | --- |
| I11 | Hypertensive heart disease |
| I12 | Hypertensive renal disease |
| I13 | Hypertensive heart and renal disease |
| I15 | Secondary hypertension |

**Stroke**

| I60 | Subarachnoid hemorrhage |
| --- | --- |
| I61 | Intracerebral hemorrhage |
| I62 | Other nontraumatic intracranial hemorrhage |
| I63 | Cerebral infarction |
| I64 | Stroke, not specified as hemorrhage or infarction |

**Ischemic heart disease**

| I20 | Angina pectoris |
| --- | --- |
| I21 | Acute myocardial infarction |
| I22 | Subsequent myocardial infarction |
| I23 | Certain current complications following acute myocardial infarction |
| I24 | Other acute ischemic heart diseases |
| I25 | Chronic ischemic heart disease |

**Heart failure**

| I11.0 | Hypertensive heart disease with (congestive) heart failure |
| --- | --- |
| I13.0 | Hypertensive heart and renal disease with (congestive) heart failure |
| I13.2 | Hypertensive heart and renal disease with both (congestive) heart failure and renal failure |
| I42.0 | Dilated cardiomyopathy |
| I42.6 | Alcoholic cardiomyopathy |
| I42.7 | Cardiomyopathy due to drugs and other external agents |
| I42.8 | Other cardiomyopathies |
| I42.9 | Cardiomyopathy, unspecified |
| I50 | Heart failure |
| I50.0 | Congestive heart failure |
| I50.1 | Left ventricular failure |
| I50.9 | Heart failure, unspecified |

**Other forms of heart disease**

| I48 | Atrial fibrillation and flutter |
| --- | --- |
| I44 | Atrioventricular and left bundle-branch block |

**Peripheral vascular disease**

| I17.0 | Atherosclerosis |
| --- | --- |
| I17.1 | Aortic aneurysm and dissection |
| I17.2 | Other aneurysm and dissection |
| I17.3 | Other peripheral vascular diseases |
| I17.4 | Arterial embolism and thrombosis |
| I17.7 | Other disorders of arteries and arterioles |

**Diabetes**

| E10 | Type 1 diabetes mellitus |
| --- | --- |
| E11 | Type 2 diabetes mellitus |
| E12 | Malnutrition-related diabetes mellitus |
| E13 | Other specified diabetes mellitus |
| E14 | Unspecified diabetes mellitus |

**Chronic kidney disease**

| BJFD2 | Dialysis in chronic kidney disease |
| --- | --- |
| E10.2 | Type 1 diabetes mellitus with kidney complications |
| E11.2 | Type 2 diabetes mellitus with kidney complications |
| E12.2 | Malnutrition-related diabetes mellitus with kidney complications |
| E13.2 | Other specified diabetes mellitus with kidney complications |
| E14.2 | Unspecified diabetes mellitus with kidney complications |
| G62 | Congenital obstructive defects of renal pelvis and congenital malformations of ureter |
| I12 | Hypertensive chronic kidney disease |
| I13 | Hypertensive heart and chronic kidney disease |
| N00 | Acute nephritic syndrome |
| N01 | Rapidly progressive nephritic syndrome |
| N02 | Recurrent and persistent hematuria |
| N03 | Chronic nephritic syndrome |
| N04 | Nephrotic syndrome |
| N05 | Unspecified nephritic syndrome |
| N06 | Isolated proteinuria with specified morphological lesion |
| N07 | Hereditary nephropathy, not elsewhere classified |
| N08.3 | Glomerular disorders in diabetes mellitus |
| N08.4 | Glomerular disorders in other endocrine, nutritional, and metabolic diseases |
| N08.5 | Glomerular disorders in systemic connective tissue disorders |
| N11 | Chronic tubulo-interstitial nephritis |
| N11.0 | Nonobstructive reflux-associated chronic pyelonephritis |
| N11.8 | Other chronic tubulo-interstitial kidney disease |
| N12 | Tubulo-interstitial nephritis |
| N15.0 | Balkan nephropathy |
| N16.2 | Renal tubulo-interstitial disorders in blood diseases and disorders involving the immune mechanism |
| N16.3 | Renal tubulo-interstitial disorders in metabolic diseases |
| N16.4 | Renal tubulo-interstitial disorders in systemic connective tissue disorders |
| N18 (1–5+9) | Chronic Kidney Disease |
| N19 | Unspecified kidney failure |
| N26 | Unspecified contracted kidney |
| N27 | Small kidney of unknown cause |
| N28 | Other disorders of kidney and ureter, not elsewhere classified |
| N29 | Other disorders of kidney and ureter in diseases classified elsewhere |
| Q60 | Renal agenesis and other reduction defects of kidney |
| Q61 | Cystic kidney disease |
| Q62 | Congenital obstructive defects of renal pelvis and congenital malformations of ureter |
| Q63 | Other congenital malformations of kidney |
| Q64 | Other congenital malformations of urinary system |
| Z94.0 | Kidney transplant status |
| Z99.2 | Dependence on renal dialysis |
