# Supplementary material for: Recognition, treatment, and control of hypertension in the Danish population-based Lolland-Falster Health Study
Source: Eur J Public Health. 2026 Jul 9;36(4):ckag117. doi: 10.1093/eurpub/ckag117 (PMC13349664; doi:10.1093/eurpub/ckag117)
Supplement: ckag117_Supplementary_Data [file ckag117_supplementary_data.zip › ejph-2026-02-om-0197-File007.docx]

| **Table S3. Guideline-defined criteria used in the cardiovascular risk stratification algorithm for assessment of antihypertensive treatment indication** | |
| --- | --- |
| **Category** | **Definition** |
| **Cardiovascular disease** | Includes cerebrovascular disease, heart failure with preserved ejection fraction, peripheral vascular disease, and advanced retinopathy (fundus hypertension grades III–IV). |
| **Chronic kidney disease** | Defined as estimated glomerular filtration rate < 30 mL/min/1.73 m² or urine albumin–creatinine ratio > 300 mg/g |
| **Risk factors** | Male sex; smoking; dyslipidemia (total cholesterol > 4.9 mmol/L, LDL cholesterol > 3.0 mmol/L, HDL cholesterol < 1.0 mmol/L in men or < 1.2 mmol/L in women, or triglycerides > 1.7 mmol/L); age > 55 years for men or > 65 years for women; abdominal obesity (waist circumference ≥ 102 cm in men or ≥88 cm in women); body mass index ≥ 30 kg/m². |
| **Asymptomatic organ damage** | Pulse pressure (systolic blood pressure minus diastolic blood pressure) ≥ 60 mmHg; estimated glomerular filtration rate 30–60 mL/min/1.73 m²; urine albumin–creatinine ratio 30–300 mg/g. |
| *These definitions are specific to the cardiovascular risk stratification and treatment algorithm applied in this study and are based on guideline criteria; they may differ from general definitions used elsewhere in the manuscript.* | |
